# Asymptomatic Severe Vagal and Sympathetic Cardiac Denervation in Holmes-Adie's Syndrome

**DOI:** 10.1155/2017/4919758

**Published:** 2017-03-27

**Authors:** B. Estañol, R. C. Callejas-Rojas, S. Cortés, R. Martínez-Memije, O. Infante-Vázquez, G. Delgado-García

**Affiliations:** ^1^Laboratory of Clinical Neurophysiology, National Institute of Medical Sciences and Nutrition, Mexico City, Mexico; ^2^Department of Electromechanical Instrumentation, National Institute of Cardiology, Mexico City, Mexico; ^3^Department of Internal Medicine, University Hospital, Autonomous University of Nuevo León, Monterrey, NL, Mexico

## Abstract

A 40-year-old woman was found to have bilateral Adie's pupils and generalized muscle stretch areflexia. She did not have orthostatic hypotension but, in an ECG strip in the office, she appeared to have an almost fixed heart rate. We thus studied the heart rate variability (HRV) and the systolic blood pressure variability (SBPV) in supine and standing position and also during rhythmic breathing. We found a decreased HRV in the time domain with very low standard deviation in supine and standing position and during rhythmic breathing. HRV in the frequency domain was low with a decrease in the absolute power of HF and LF and a decrease in the sympathovagal balance in supine and standing positions. SBPV in the time and frequency domains was found to be normal. This patient with Holmes-Adie syndrome had an asymptomatic severe loss of HRV and a preserved SBPV. The global decrease in the HRV in the time and frequency domains indicated that she had both vagal and sympathetic cardiac denervation, whereas the preserved SBPV suggested normal innervation of the blood vessels.

## 1. Introduction

Cardiovascular autonomic dysfunction in patients with Holmes-Adie syndrome (HAS) has been recognized since Croll and Duthie's first description (1935) of orthostatic hypotension (OH) in one of these patients [1]. This report was followed by others [2, 3]. Johnson et al. were the first to extensively study the baroreceptor function of two patients with HAS and OH. Their findings suggested a block of afferent nerves from arterial baroreceptors [4]. Other investigators have subsequently reported conclusions consistent with afferent baroreceptor dysfunction [5].

Cardiovascular autonomic function in patients with HAS but without OH has been only studied in depth in a few reports [5–7]. Based on previous small studies, it has been estimated that 28 to 40% of patients with HAS also have autonomic dysfunction [8]. The definite proportion of patients with HAS and vagal or sympathetic cardiac dysfunction is nevertheless currently unknown. In one study, 4 (36%) out of 11 patients were considered to have abnormalities of parasympathetic function. They were given a battery of tests including the Valsalva maneuver, standing from lying position, and deep breathing [6]. Given the fact that the pupils have cholinergic denervation, it is reasonable to expect that the heart and other organs may also have vagal denervation. In another study, 21 (40%) out of 53 patients with HAS had evidence of autonomic dysfunction [7]. Until now, in these patients, no study has been reported on the heart rate variability (HRV) and systolic blood pressure variability (SBPV) analyzed in the frequency domain.

A patient with bilateral Adie's pupils and generalized muscle stretch areflexia was investigated to elucidate whether she also had an abnormal HRV and SBPV. Our analysis was performed in the time and frequency domains.

## 2. Case Presentation

**Figure 1:**
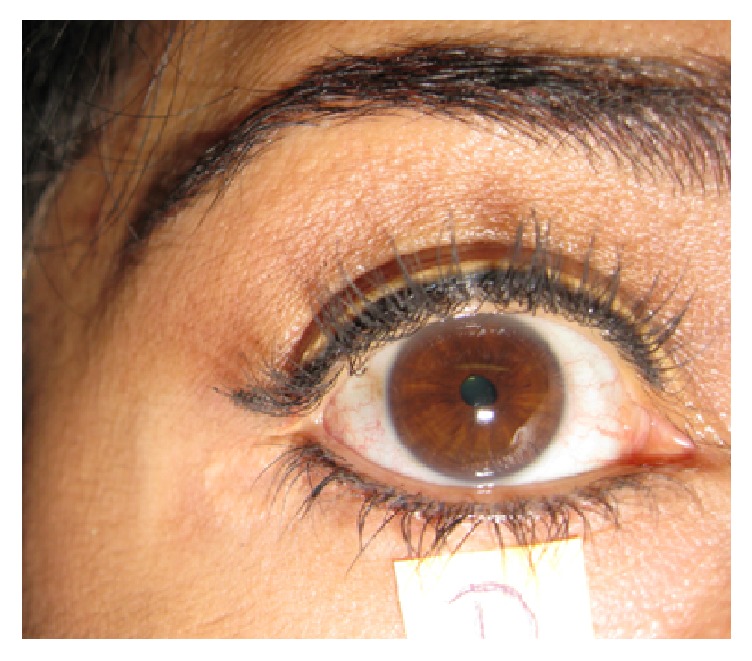
Right pupil with oval shape unresponsive to light stimulation delayed dilatation after accommodation; contracted briskly to accommodation and pilocarpine 0.125%.

A previously healthy 40-year-old woman was referred to us by an ophthalmologist who noticed anisocoria with a small right pupil and a dilated left pupil. She had no ocular symptoms and saw the ophthalmologist because of conjunctivitis. She gave a history of flu-like illness that lasted about three months; that acute illness occurred five months before the eye examination and resolved eventually without any treatment. No diagnosis was made at that time. She did not complain of difficulties with her vision and could read without difficulty. She denied fever, fatigue, dizzy spells, tachycardia, syncope, recurrent vomiting, dry mouth, rhinitis, dry eyes, constipation, or bladder symptoms. She did not complain of lack of sweating (anhidrosis) or hyperhidrosis and tolerated well changes of temperature. Her general physical examination was unremarkable. Blood pressure was 120/80 mmHg in supine and standing position, and her pulse was 65 beats per minute in supine and standing position. Neurological examination showed generalized absence of muscle stretch reflexes that could not be elicited with the Jendrassik maneuver. Several independent neurologists confirmed the absence of reflexes in the lower and upper extremities. Plantar reflexes were flexor. Romberg sign was absent; there was no weakness and no sensory ataxia. Careful sensory examination for touch, pin prick, and cold was normal. Position sense of the toes and fingers was normal. She could perceive well the vibration of a tuning fork (256 Hz) distally and proximally in both upper and lower extremities. Eye movements were normal. Her right pupil was 1.5 mm and had an oval shape, with no response to light (Figure 1); the left pupil measured about 6 mm and responded very poorly to light. The response to light of the left pupil was segmentary with a slight response of the right superior segment. Both responded poorly to accommodation although the left pupil remained slightly more miotic after an accommodative effort. The pupils contracted briskly with pilocarpine dilution of 0.125%. Brain and cervical cord MRI was normal. A five-minute ECG strip in DII appeared to have an almost fixed heart rate. The lack of significant tachycardia during standing and the ECG findings prompted the physicians to perform autonomic cardiovascular studies.

A normal sympathetic skin response (SSR) was obtained in the palms and the soles with electric stimulation of the median nerve. The latency at the palm was 1.34 seconds, and that at the soles was 1.89 seconds. The SSR could also be obtained with deep inspiration and with a cough. Four extremities, sensory and motor conduction velocities, and F responses were performed and found to be normal (Viking, Viasys, 2008).

We studied the HRV and the SBPV by capturing the RR interval and the systolic, diastolic, and mean blood pressure using Finometer® PRO (Finapres Medical Systems BV, Netherlands) under the following conditions: (1) supine at rest during a period of five minutes; (2) standing up, for five minutes, after the BP and the HR have become stabilized; and (3) during rhythmic breathing at 0.1 Hz (6 cycles per minute) during a period of five minutes. The signals were obtained with 200 Hz sampling rate with an analogic/digital resolution of 16 bits. We analyzed the five-minute time series for the RR intervals and the blood pressure time series with the BeatScope® software. The interbeat intervals time series were analyzed in milliseconds (RR intervals) and also in beats per minute (HR); the blood pressure time series were analyzed in mmHg. We performed the time domain analysis measuring (1) mean HR and SBP, (2) standard deviation of HR and SBP, (3) range of HR and SBP, (4) maximum, (5) minimum, and (6) ratio of maximum/minimum HR in the three conditions. We studied the frequency domain using the Fast Fourier Transform (MATLAB®, 1999) and obtained the following data: (1) absolute power of HF (high frequency, 0.15–0.4 Hz), (2) absolute power of LF (low frequency, 0.04–0.15 Hz), (3) sympathovagal balance (SVB = LF/HF), (4) tachograms, and (5) total spectral analysis of the HR and SBP. We also obtained the histograms and the Poincaré plots of the HR in the three conditions. These studies were performed in the morning after a light breakfast. The patients did not use anticholinergics, adrenergics, beta-blockers, coffee, or tobacco before the studies.

**Figure 2:**
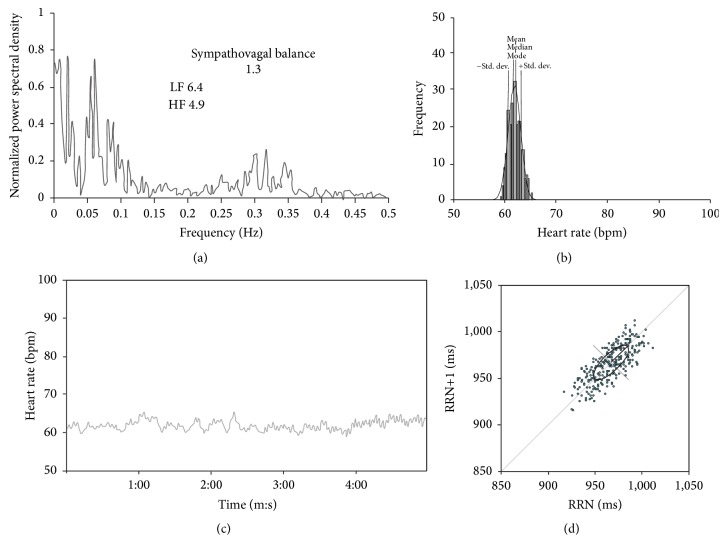
HRV in the supine position. (a) The total Fourier spectrum, (b) the histogram, (c) the tachogram, and (d) the Poincaré plot. The sympathovagal balance (SVB = LF/HF) was 1.31 versus 0.85 ± 0.3 in controls. The total power of LF was 6.4 versus 8.1 ± 2.5 in controls, and the total power of HF 4.9 versus 10.1 ± 3.6 in controls. The tachogram, the histogram, and the Poincaré plot show a loss of variability across all frequencies.

**Figure 3:**
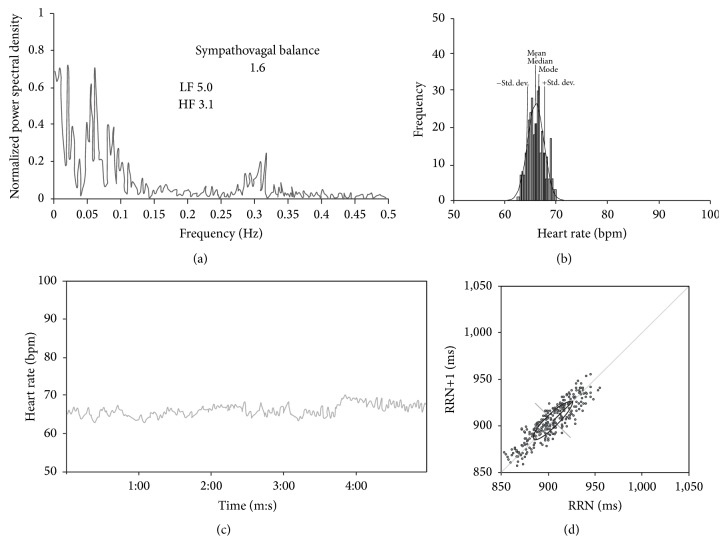
HRV in the standing position. (a) The total Fourier spectrum, (b) the histogram, (c) the tachogram, and (d) and the Poincaré plot. In (a), the sympathovagal balance is included (SVB = 1.62 versus 1.5 ± 0.6 in controls). The normal SVB seems to be due to a severe reduction of the total power of HF and a less severe reduction of LF so the ratio remained normal. The total power of LF was 5.0 versus 6.2 ± 1.9 in controls, and the total power of HF was 3.10 versus 10.1 ± 3.6 in controls. There was a severe loss of variability across all frequencies.

**Figure 4:**
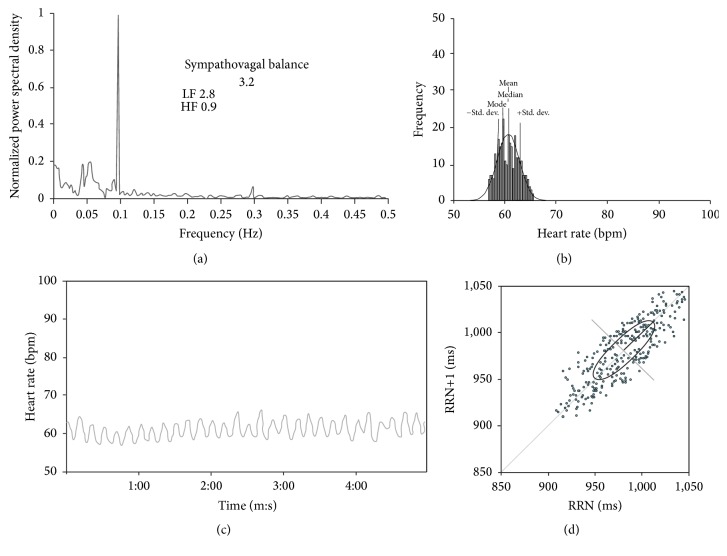
HRV during rhythmic breathing. (a) The total Fourier spectrum, (b) the histogram, (c) the tachogram, and (d) the Poincaré plot. In (a), the sympathovagal balance is included (SVB = 3.27 versus 1.83 ± 0.5 in controls); the total power of LF was 2.8 versus 4.3 ± 2.4 in controls, and the total power of HF was 0.9 versus 2.4 ± 1.2 in controls. Although some respiratory driving is present, the magnitude of the driving is abnormally decreased.

The HRV was ostensibly decreased in the time domain in the supine and standing position and also during rhythmic breathing (Figures 2, 3, and 4). The SD was markedly decreased, as were also the maximum/minimum ratio, the range, and the variation coefficient (VC) (Table 1). The analysis of the HRV in the frequency domain, in the three conditions, showed a decrease in the absolute power of LF and HF. The increase in SBV was due to the decrease of both the LF and the HF, although the HF was most severely decreased (Table 1).

In Table 2, the normal values of the SBPV are depicted in the time domain together with the patient's values. The patient's values are within normal limits which suggest normal innervation of the blood vessels. In Table 2, the values in the frequency domain in normalized units are shown in the three conditions; the patient's values are within normal limits.

## 3. Discussion

Elaborate studies of autonomic cardiovascular function of patients with HAS without OH are limited [5–7] and, as far as we know, none of these have assessed cardiovascular variability (HRV and SBPV) in the frequency domain. HRV in our patient was markedly decreased, as shown in the analysis in the time and frequency domains, whereas the SBPV was found to be normal. HRV was affected in several ways. There was a very low standard deviation either in the supine or in the standing position. The respiratory drive of HR was also comparatively reduced. These findings suggest parasympathetic cardiac dysfunction. The LF component of HRV was decreased in all three conditions, and it was even comparatively decreased when standing. This component has been traditionally described as a measure of sympathetic activation, a component of activity in SVB, or a measure of sympathetic activity [9]. However, there is currently no agreement on whether this component is a reliable index of sympathetic activity [10–12], and there is also a growing body of evidence suggesting that it is not a specific marker for cardiac sympathetic tone [10, 11]. Sympathetic dysfunction was somewhat unexpected because the cholinergic denervation of the pupils led us to think that vagal denervation would be the sole abnormality in the HRV. The absence of OH, as well as the normal SBPV, suggests that the blood vessels of this patient had normal innervation. Other sympathetic innervations have been found to be abnormal in patients with HAS [13]. Anhidrosis is particularly prominent in patients with Ross syndrome. This syndrome is characterized by the triad of areflexia, tonic pupils, and segmental anhidrosis [14]. Anhidrosis was not present in our patient but sympathetic cholinergic denervation to the sudomotor glands is the cause of the anhidrosis in these patients with Ross syndrome. Despite normal cardiovascular reflex tests, cardiac sympathetic denervation has been demonstrated by MIBG-SPECT in a patient with Ross syndrome [15]. The findings in our patient suggest that some patients with HAS may have vagal and sympathetic cardiac dysfunction. Future studies with a greater number of patients are nevertheless necessary.

## Figures and Tables

**Table 1:** Heart rate variability in the time and frequency domains.

	Supine	Standing	Rhythmic breathing
Mean HR	61.9	66.3	60.8
SD	1.2 (normal: 3.7 ± 1.5)	1.5 (normal: 5.3 ± 2)	2.1 (normal: 7.1 ± 2.7)
Range	6.2 (normal: 23 ± 9)	7.3 (normal: 27 ± 9)	8.8 (normal: 32 ± 9)
Max HR	65.3	70.1	65.7
Min HR	59.1	62.8	56.8
Max/min	1.1 (normal: 1.4 ± 0.2)	1.1 (normal: 1.45 ± 0.2)	1.1 (normal: 1.59 ± 0.2)
VC (%)	1.9 (normal: 5.6 ± 2.5)	2.3 (normal: 6.6 ± 2.9)	3.4 (normal: 10.5 ± 4.5)
LF total power (normalized)	6.4 (normal: 8.1 ± 2.5)	5.0 (normal: 6.2 ± 1.9)	2.8 (normal: 4.3 ± 2.4)
HF total power (normalized)	4.9 (normal: 10.1 ± 3.6)	3.1 (normal: 4.9 ± 2.6)	0.9 (normal: 2.4 ± 1.2)
SVB	1.3 (normal: 0.85 ± 0.3)	1.6 (normal: 1.5 ± 0.6)	3.1 (normal: 1.83 ± 0.5)

Normal values for our laboratory in 30 healthy controls.

**Table 2:** Systolic blood pressure variability in the time and frequency domains.

	Supine	Standing	Rhythmic breathing
Mean SBP (mmHg)	108	107	109
SD	4.8 (normal: 5.6 ± 2.1)	3.1 (normal: 5.7 ± 1.4)	7.0 (normal: 7.1 ± 1.6)
Range	22 (normal: 28.8 ± 8.4)	18 (normal: 30.7 ± 7.7)	32 (normal: 35.9 ± 6.1)
Max SBP	122	117	128
Min SBP	100	99	96
Max/min	1.2 (normal: 1.3 ± 0.1)	1.1 (normal: 1.3 ± 0.1)	1.3 (normal: 1.4 ± 0.1)
VC (%)	4.5 (normal: 5.4 ± 2.3)	2.9 (normal: 5.3 ± 1.3)	6.4 (normal: 6.8 ± 2.0)
LF total power (normalized)	9.4 (normal: 6.7 ± 2.8)	12.9 (normal: 6.9 ± 2.9)	4.1 (normal: 6.4 ± 2.2)
HF total power (normalized)	7.3 (normal: 3.9 ± 2.4)	10.2 (normal: 3.8 ± 2.2)	1.0 (normal: 2.6 ± 1.5)
SBV	1.3 (normal: 2.0 ± 0.7)	1.3 (normal: 2.2 ± 0.8)	4.2 (normal: 2.7 ± 0.7)

Normal values for our laboratory in 30 healthy controls.
